# Effects of Supraphysiological Doses of Sex Steroids on Wheel Running Activity in Mice

**DOI:** 10.4172/2157-7536.1000110

**Published:** 2012-07-09

**Authors:** Robert S Bowen, Amy M Knab, Alicia Trynor Hamilton, Jennifer R McCall, Trudy L Moore-Harrison, J Timothy Lightfoot

**Affiliations:** 1 Science and Mathematics Division, Truett-McConnell College, Cleveland, GA 30528, USA; 2 Department of Kinesiology, University of North Carolina Charlotte, Charlotte, NC 28223, USA; 3 Appalachian State University, Human Performance Laboratory, North Carolina Research Campus, Kannapolis, NC 28081, USA; 4 Molecular Biology and Microarray Core Facility, Cannon Research Center, Carolinas Medical Center, Charlotte, NC, 28203, USA; 5 Department of Biology, University of North Carolina Charlotte, Charlotte, NC, 28223, USA; 6 Center of Marine Science, University of North Carolina Wilmington, Wilmington, NC, 28409, USA; 7 Department of Health and Kinesiology, Texas A&M University, College Station, TX 77845, USA

**Keywords:** Sex hormones, Physical activity, 17β-Estradiol, Testosterone

## Abstract

The regulatory mechanisms of physical activity are postulated to include environmental and biological/genetic factors. In particular, the sex steroids appear to have profound effects on wheel running in rodents. The purpose of this project was to investigate the effects of 17β-estradiol and testosterone on wheel running distance, duration, and speed in male and female C57BL/6J mice. The mice (N=46) were provided free access to running wheels interfaced with computers to track daily running distance, duration, and speed. Activity was assessed at baseline in intact mice, after surgical gonadectomy, and after replacement with either 17β-estradiol or testosterone. Upon removal of the gonads, physical activity levels were significantly reduced in both males and females. Distance (10–30% of baseline) and duration (20–47% of baseline) measures were most affected by the loss of endogenous steroids, while running speed (60–77% of baseline) though significantly reduced-decreased by a much lower magnitude. Testosterone replacement fully recovered running distance, duration, and speed to pre-surgical levels in both sexes (100% of baseline). Distance (30–42% of baseline) and duration (43–47% of baseline) were partially recovered by 17β-estradiol, but not to baseline levels. Speed (100% of baseline) was fully recovered by 17β-estradiol replacement in males and females. This study suggests that physical activity in mice is affected by endogenous steroids and can be altered by exogenous steroid replacement. The differences in the recovery abilities of 17β-estradiol and testosterone suggest that both estrogenic and androgenic pathways may be involved to variable degrees in activity regulation.

## Introduction

Physical inactivity enhances the risk of many diseases including obesity, diabetes, many types of cancer, and heart disease [1]. The US health care system is excessively burdened by hypokinetic related diseases, resulting in reduction in service and care. Furthermore, quality of life parameters are significantly degraded as inactivity induced diseases progress. To understand the mechanisms inducing physical inactivity within the human population, efforts to elucidate the biological and genetic factors that alter either the motivation or ability to partake in increased activity are necessary.

The sex steroids have previously been shown to influence physical activity in rodents and may be important biological factors regulating activity levels. Gorzek et al. [2] altered 17β-estradiol levels in young female mice by surgically removing their ovaries followed by replacement in a capsulated form. The study showed a significant decrease in voluntary wheel running distance following ovariectomy and a recovery back to pre-surgery levels when capsulated 17β-estradiol was administered. A similar response has been shown in male rats after castration [3]. Several other studies report comparable effects in various rodent species [4–8]; however, with the exception of the study by Gorzek et al. [2], these studies were conducted in the 1920s prior to the discovery and purification of many of the chemicals involved in activity regulation. The results from these studies, though unique and novel for the early parts of the 20th century, are outdated and require revision using newly available delivery techniques, measurement apparatuses, and purified steroid samples.

In a seminal study, Roy and Wade [9] administered dihydrotestosterone propionate, a non-aromatizable form of testosterone, to castrated male rats and found no significant change in activity levels suggesting that an estrogen-aromatase dependent mechanism was responsible for activity regulation by the sex steroids. Additionally, using estrogen receptor α (ERα) and β (ERβ) knock-out mice, Ogawa et al. [10] demonstrated alterations in wheel running via ERα, but not ERβ pathways. Finally, it has been hypothesized that changes to activity in murine systems due to changes in sex steroid levels may be due in part to undiscovered non-genomic effects and/or intricate interactions between estrogens, ERα, and dopaminergic neurons [11].

The purpose of this study was to systematically remove and replace both 17β-estradiol and testosterone in male and female mice allowing comparisons to be made between the steroids and sexes in the regulation of running wheel activity. Alteration in running distance or number of wheel revolutions has been the flagship measure defining physical activity levels in mouse models [2–3,5–8,12–14]; newer techniques now allow running duration and speed to be quantified. Thus, a secondary purpose of this study was to evaluate the changes in running distance, duration, and speed in a murine model of physical activity under minimal and supra-physiological levels of circulating sex steroids.

## Materials and Methods

### Animals

This project conformed to standards of humane animal care and received approval from the UNC Charlotte Institutional Animal Care and Use Committee prior to initiation. C57BL/6J inbred mice (Jackson Laboratory, Bar Harbor, ME) were used in this study due to their prevalent use in the scientific literature and because of their genetic homogeneity. Twenty-three male and 23 female mice were initially used in this study; however, five mice (male=1; female=4) showed signs of distress following the surgical gonadectomy procedures and were euthanized for humane purposes resulting in a total cohort of 41 animals for the remainder of the study. Prior to the start of this project, mice were group housed three to four per cage until they reached approximately 9 weeks of age. The mice were then individually housed and provided unlimited access to running wheels for the duration of the study. Whereas mice reach their activity zenith between 9 and 12 weeks of age [15] this study encompassed the most active parts of the lifespan. Through the entirety of the study, the mice were housed under a 12:12hr light: dark cycle initiating daily at 6am. Free access to water and standard mouse chow (Harlan Teklad, Madison, WI) was provided throughout the study. The chow provided to the mice during this study was not phytoestrogen-free. Several authors [16–20] have shown that phytoestrogens do not increase activity in gonadectomized mice; therefore the use of phytoestrogen-free food did not affect activity levels in this study.

### Experimental procedures

**Figure 1:**
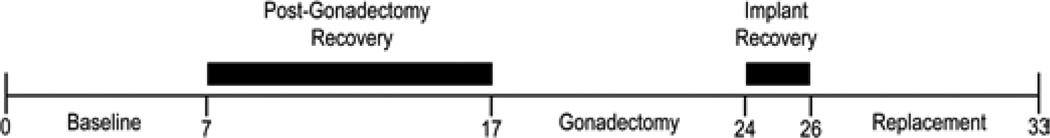
Experimental timeline (in days) for study assessing physical activity differences in C57BL/6J mice under various circulating sex steroid levels including normal physiological levels at baseline, low levels during gonadectomy phase, and elevated levels during replacement phase. Baseline, gonadectomy, and replacement indicate periods when wheel running activity was measured. Dark black bars indicate the recovery periods immediately following the gonadectomy surgery (beginning of day 8) and silastic capsule implantation surgery (beginning of day 24).

The timeline for this project is displayed in Figure 1. Each mouse was randomly assigned to either an experimental group or a control group. This random assignment was stratified by sex and by the initial housing scheme in order to ensure that previous group housing effects would be minimized. After separation into individual cages, each mouse was supplied a running wheel. Wheel running distance, duration, and speed represented physical activity levels and were monitored under three experimental treatments including at baseline, after gonadectomy, and with supraphysiological steroid replacement (detailed below).

After an initial seven-day period to assess baseline wheel running, gonadectomy surgeries (detailed below) were performed to reduce circulating steroid levels. The control groups received sham surgeries. A 10-day recovery period, without wheels, allowed the surgical wounds to heal and remaining circulating steroids to clear from the system. After this recovery period, the wheels were replaced in the cages. Wheel running activity was then tracked for an additional seven days.

Next, the implant surgeries were completed and followed by two days of recovery. Activity was monitored for seven days. Silastic capsules (detailed below) containing 17β-estradiol were implanted in eight females and nine males. Silastic capsules containing testosterone were implanted in eight females and ten males. The animals in the control groups were given empty implants. The two-day recovery period allowed the animals to recover from surgical wounds and the silastic implants to deliver steroid into the bloodstream [21]. After recovery, the mice were re-exposed to running wheels and their physical activity levels were monitored for seven days. At the end of each seven-day data collection period, body masses and percentage body fat measures were completed. A PIXImus 2.10 (Lunar, Madison, WI) was used to collect the percentage body fat measurements via dual energy x-ray absorptiometry.

### Measurement of wheel-running activity

Physical activity was measured by determining daily distance, duration, and speed of wheel running using standard protocols [22,23]. In brief, running wheels were mounted to the cage tops of standard rat cages and were equipped with a cycling computer (BC500, Sigma Sport, Batavia, IL) to record running distance and duration. Running wheels had a 450 mm circumference and a 40 mm wide, solid running surface. Running distance and duration data were collected on a daily basis in the morning and average daily running speed was calculated from the corresponding distance and duration measures. The sensor and magnet alignment and freeness of the wheel were checked daily and adjusted as needed.

### Surgical procedures

Twenty males and twenty females received orchidectomies or ovariectomies. The remaining six mice (3 males; 3 females) acted as control animals and underwent sham surgical procedures. A preemptive dose (0.05 mg·kg^−1^) of buprenorphine was administered via intraperitoneal injection approximately 30 minutes prior to the gonadectomy procedures. All procedures were performed under light isoflurane anesthesia with a 300 ml·min^−1^ oxygen flow rate. Incisions were made under sterile conditions (10% betadine followed by 70% alcohol) with sterile surgical tools.

The gonadectomy surgery performed depended upon the sex of the mouse. Orchidectomy surgeries were preformed on the male mice and were initiated by making a small access incision in the skin directly proximal to the scrotal sac. Additional incisions were made in the fascia on either side of the scrotal sac. Slight pressure was applied just above the incision sites to expose the testes. Both the testes and epididymis were excised and discarded. The incision wound in the skin was closed with a surgical staple. Bilateral ovariectomies were preformed on the female mice. A small incision was made in the skin directly above the lumbar region. A small pocket was developed between the skin and muscle to allow unrestricted access to the animal’s dorsolateral region. Small incisions were made in the fascia approximately 5 mm on either side of the spine just proximal to visible white fatty tissue. Each ovary was exposed and excised. The skin wound was closed with a surgical staple. The sham procedures performed on the control animals were identical to the procedures described above minus the excision of the sex organs.

### Replacement procedures

Two sets of implants were developed to release sex steroids into the bloodstream of the mice based on diffusion. The silastic implants were produced similar to the technique of Cohen and Milligan [21], except that dry powder without arachis oil was packed into silastic tubing. A 10 mm section of the silastic tubing (Dow Corning, Midland, MI) with an outer diameter of 2.16 mm and inner diameter of 1.02 mm was packed with either powder testosterone or powder 17β-estradiol (Sigma-Aldrich, St. Louis, MO). The ends of the tubing were covered with a small bead of weatherproof silicone glue. Each implant was washed in 70% alcohol for one minute and rinsed in deionized water. After washing, the implant was patted dry and stored in Eppendorf tubes at room temperature under dark, dry conditions. The implants for the control animals were prepared in the same way, but were left empty. Surgeries to implant silastic capsules to replace the sex steroids were preformed during the later stages of this project. A small incision was made on the lateral aspect of the neck. A cavity about 15 mm in depth and width was developed between the skin and muscle tissue. Forceps were used to insert a 10 mm long silastic implant into the cavity. The incision wound was closed with a surgical staple.

### Sex steroid assays

The current project was performed in conjunction with two other related projects. Blood samples were taken on regular two week intervals during the studies and at the end of each project. Blood samples taken during the project were completed on live animals. The mice were immobilized in a decapicone bag with slots for the hind limbs. The medial aspects of the hind limbs were cleaned and blood was sampled from the saphenous vein via venipuncture. Plasma was retrieved after cold centrifugation and individual samples (n=3) from each experimental condition were pooled. The blood sampled at the end of experiment was taken directly from the inferior vena cava. These samples were allowed to clot at room temperature and were then centrifuged. The serum was also pooled (n=3) based on common inclusion in a given experimental condition. The pooled samples were extracted in ethyl acetate (Sigma Aldrich, St. Louis, MO) that was then evaporated. The residue was re-suspended in steroid free serum (IBL America, Minneapolis, MN).

Testosterone (ng·ml^−1^) and 17β-estradiol (pg·ml^−1^) was measured via ELISA (IBL America, Minneapolis, MN) per the manufacturer’s instructions. The data were assessed for errors and outliers and adjusted accordingly. Data points with high variation between duplicate measures and considerable deviation from the condition mean were eliminated from analysis. Unfortunately, viable blood samples were not obtained from the female cohort, but vaginal smears and inspection of the uterine horns were completed to evaluate the effectiveness of each experimental intervention.

### Statistical analysis

Distance, duration, and speed were averaged for seven days per experimental treatment (i.e. baseline, gonadectomized, and replaced). Unpaired-sample t-tests were utilized to evaluate the overall mean differences attributable to sex. Separate two-way (group by treatment) analysis of variance (ANOVA) calculations were used to assess differences between the treatment levels for each physical activity variable. A three-way ANOVA was used to compare the body composition measures between groups, sexes, and experimental periods. Tukey’s HSD *post-hoc* tests were used if the main effects or interactions from the initial ANOVA reached significance. The alpha value was set *a priori* to 0.05.

## Results

### Male mice

**Figure 2:**
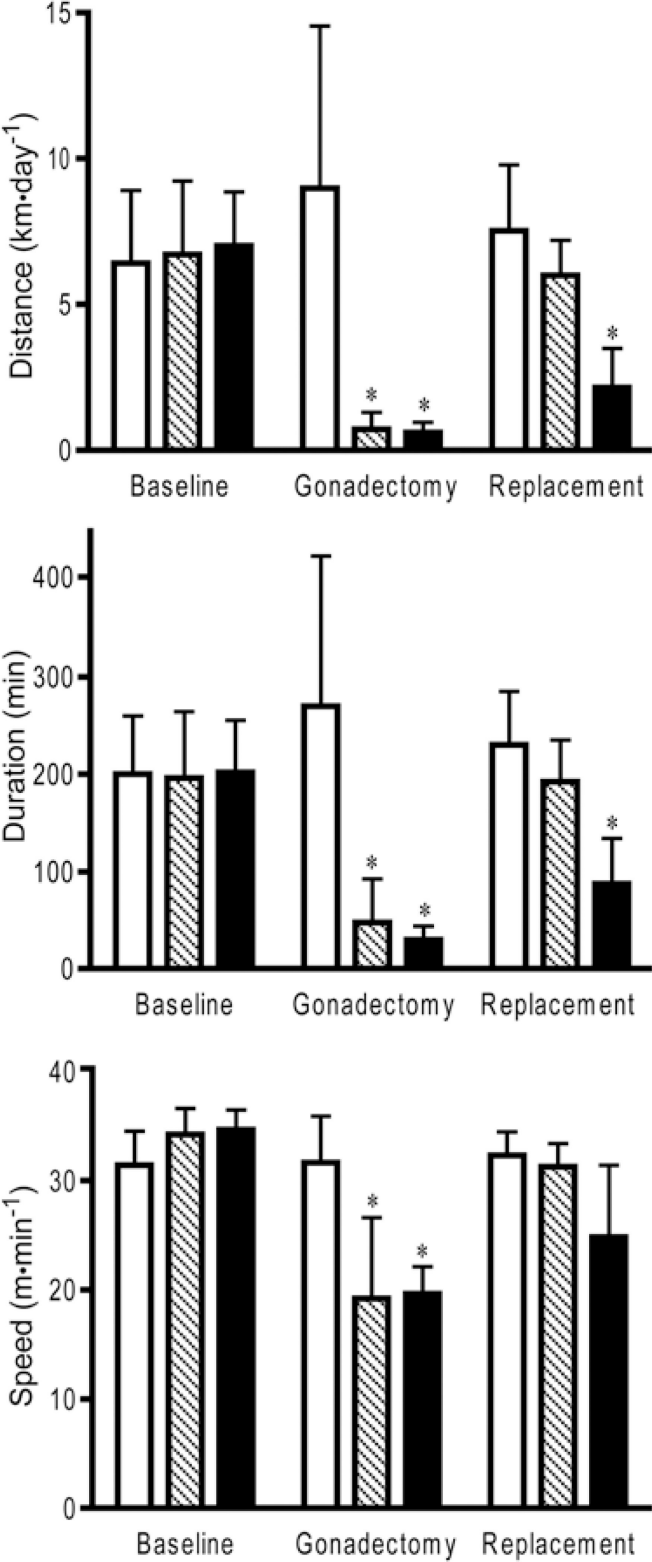
Male C57BL/6J mice wheel running distance, duration, and speed under normal physiological condition (Baseline), with low circulating steroid levels (Gonadectomy), and with artificially elevated steroid levels (Replacement). White bars=sham controls; Hatched bars=testosterone during replacement period; Black bars=17β-estradiol during replacement period; *=significantly different from sham controls and baseline treatments.

While no difference was noted between the treatment groups during the baseline period (i.e. before gonadectomy), physical activity patterns of the male mice were markedly altered by removal and replacement of the sex steroids (Figure 2). After orchidectomy, both the testosterone and 17β-estradiol groups ran significantly less (10% of baseline, *p*<0.05) than the sham group and compared to baseline measurements. Replacement of testosterone recovered the activity pattern back to baseline and sham levels (90% of baseline). Replacement of 17β-estradiol partially recovered running distance (31% of baseline), but the distance remained significantly less (*p*<0.05) than baseline and sham values. Wheel running duration mirrored the distance results. At baseline, running durations were similar among the different experimental groups. Orchidectomy significantly reduced (19% of baseline) daily running duration compared to the sham group and baseline values (*p*<0.05). Testosterone replacement recovered duration to baseline levels (97% of baseline) while 17β-estradiol replacement failed to recover running duration to baseline levels (44% of baseline). Average wheel running speed was significantly influenced (59% of baseline) by sex gland removal (*p*<0.001) and recovered to near baseline after replacement. Running speed was significantly recovered during testosterone replacement (93% of baseline). 17β-estradiol replacement increased running speed to near baseline levels (74% of baseline), but did not recover running speed by the same magnitude as testosterone replacement.

### Female mice

**Figure 3:**
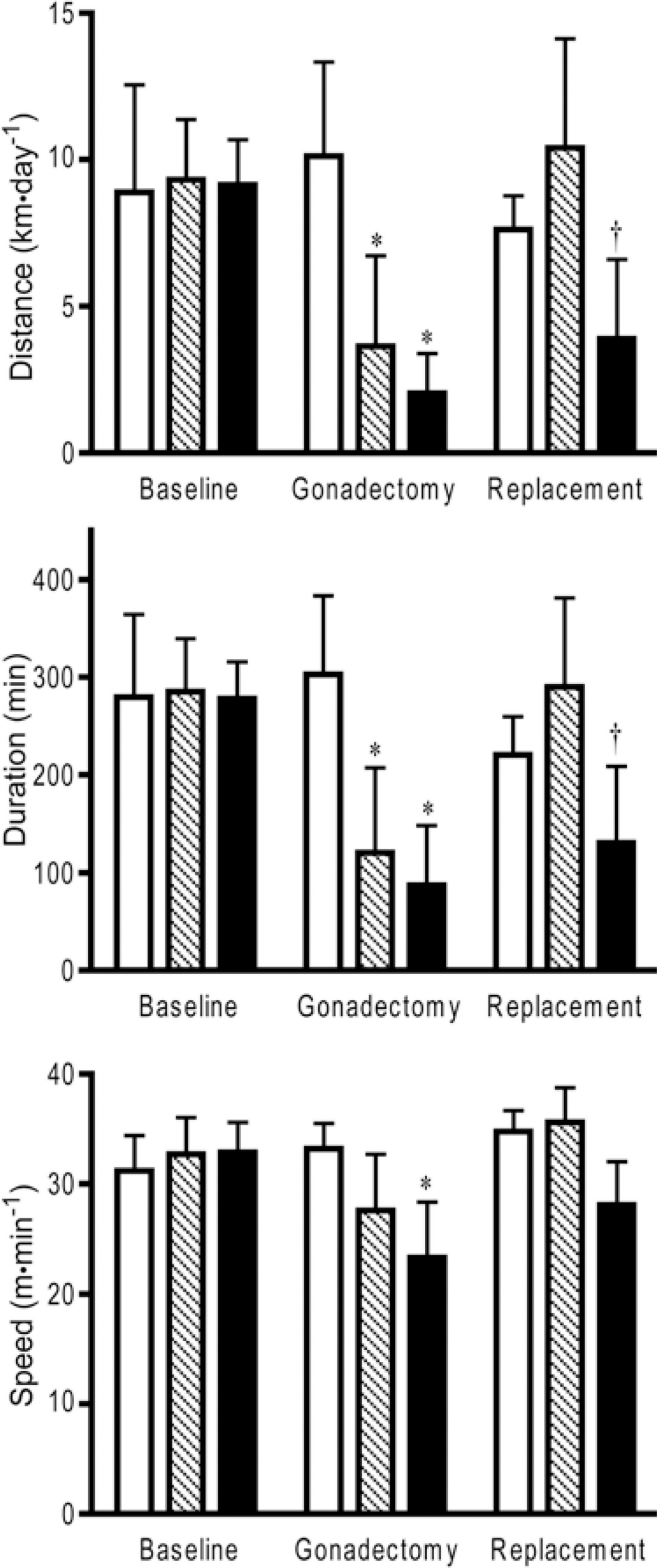
Female C57BL/6J mice wheel running distance, duration, and speed under normal physiological condition (Baseline), with low circulating steroid levels (Gonadectomy), and with artificially elevated steroid levels (Replacement). White bars=sham controls; Hatched bars=testosterone during replacement period; Black bars=17β-estradiol during replacement period; *=significantly different from sham controls and baseline treatments; †=significantly different from baseline.

Without regard to the steroidal or surgical conditions of the mice (all female data points compared to all male data points), the females ran farther (*t*=3.87; *p*<0.001), longer (*t*=4.06; *p*<0.001), and faster (*t*=2.24; *p*<0.05) than their male counterparts. However, similar to the males, removal and replacement of the sex steroids altered the physical activity patterns of the female mice (Figure 3). The female mice did not show differences in the running pattern among the experimental groups at baseline. As in the male mice, both the surgical and replacement interventions influenced running distances. After ovariectomy, wheel running distance was partially reduced (31% of baseline, *p*<0.05); running activity was reduced less in females than males after surgery. Administration of testosterone fully recovered running distance (114% of baseline), while replacement of 17β-estradiol only slightly recovered running distance (43% of baseline). After removal of the ovaries, running duration was significantly reduced (37% of baseline, *p*<0.05). Testosterone increased running time to the highest levels recorded during the experiment (103% of baseline). After replacement of 17β-estradiol, wheel running duration remained significantly different compared to baseline values (46% of baseline, *p*<0.05). Running speed was significantly reduced after removing the ovaries (79% of baseline, *p*<0.05) in the 17β-estradiol treatment group, but not in the testosterone group (85% of baseline). Testosterone administration increased (107% of baseline) running speed slightly above the baseline and sham values. Replacement with 17β-estradiol slightly (86% of baseline) increased running speed.

### Body composition

The body mass (g) and body fat (%) measures are summarized in Table 1. The body masses varied between sexes and across the experimental periods. Males (25.1 ± 1.9) weighed significantly more than females (20.9 ± 2.0) throughout the study. The body mass increased significantly across all three periods (baseline: 21.8 ± 2.9; after gonadectomy: 22.5 ± 2.3; with replacement: 25.0 ± 2.6). The percentage of body fat was also altered across experimental periods; percentage body fat was significantly lower during replacement (11.14 ± 0.90) compared to baseline (14.13 ± 2.04) and gonadectomy (13.11 ± 1.20).

### Implant efficacy

The functionality of silastic implants to deliver sex steroids in rodents has been previously shown and is a common technique in the endocrinology literature [18,21,24–28]. Direct measurement of sex steroid blood plasma levels in the mice observed during study is difficult due to the necessary volume restrictions placed on survival blood draw techniques. Viable blood samples were obtained from male mice and ELISA data were summarized in Table 2. The function of the implants and surgical success were confirmed via several direct observations in the female mice. First, the female mice receiving implants presented with visibly larger uterine horns when compared to control animals, suggesting circulating steroids were present in these mice [29]. Secondly, the content of vaginal smears taken from individuals with steroid implants, were dominated by the presence of cornified epithelial cells, which also are indicative of estrogen replacement [29]. Thirdly, the vaginal content of the mice after gonadectomy, but prior to implantation of silastic capsules was void of cellular debris. Thus, the successful use of silastic implants by past researchers to deliver steroid compounds to rodents [21] and the direct observations made during this study suggest effective delivery of 17β-estradiol and testosterone to the present cohort of mice.

In addition to the aforementioned observations, semi-quantitative measures of steroid release were made. Each implant contained approximately 3600 µg of powder steroid prior to placement in a mouse. Accurate post usage measurement of steroid containing devices is difficult due to the absorption of extraneous bodily fluids as the steroid moves into circulation. With the use of the current method of steroid delivery it was evident through direct observation that during the seven day period approximately half (1800 µg) of the powder had exited the implant upon retrieval of the capsule from the mice. Based on these observations it was estimated that between 200 and 300 µg was released from the capsules per day over the seven-day period. Comparing these data to the data of Cohen and Milligan [21], the capsules used in this study induced supraphysiological levels of the steroid in circulation as these authors demonstrated a five-fold increase in vaginal smear response and a nine-fold increase in uterine weight after an eight day exposure to silastic capsules containing 17β-estradiol (100 µg·ml^−1^). This method of steroid delivery was chosen to ensure adequate delivery of the steroids into the blood and the tissues involved in activity regulation. It has previously been shown that the blood plasma levels of the sex steroids are not equal to the levels of the steroids found in other tissue areas including the brain [30].

## Discussion

In this study, running distance, duration, and speed were significantly reduced following surgical removal of the gonads in male and female C57BL/6J inbred mice. After the diminution of wheel running activity, attempts to recover activity levels via exogenous testosterone and 17β-estradiol produced variable magnitudes of recovery. Testosterone’s propensity to boost the activity pattern in mice to normal levels after gonadectomy (101% of baseline; average across all wheel running characteristics) was evident in both males and females, surprising given the limited and contradictory [9] literature available regarding testosterone replacement. As surprising, were the somewhat limited effects of administered 17β-estradiol which resulted in only partial recovery of the activity patterns of both sexes (54% of baseline; average across all wheel running characteristics). Interestingly, the present study indicates that both testosterone and 17β-estradiol influenced activity primarily by modulating running distance and duration as opposed to speed. Thus, while administration of estrogens has been shown previously to recover activity levels to some extent [2], these are the first data to suggest an equal or higher activity recovery level with testosterone administration.

### Running wheel activity

This study evaluated physical activity patterns as a multifaceted character because running duration and speed have not been investigated in the previous sex steroid related literature. These physical activity characteristics have been suggested to contain a significant genetic component [31,32] but the mechanisms are yet to be fully delineated. Given that the treatment we used was replacement of the sex steroids, the design of the present study allowed for further understanding of the interactions of the sex steroids and these physical activity characteristics. The novel aspect of the current study is that it examined both sexes under individual influences of both an estrogenic and androgenic compound via gonadectomy and replacement procedures.

The majority of activity studies present in the literature have used number of wheel revolutions or running distance (revolutions multiplied by wheel circumference) to evaluate changes to physical activity patterns and have mostly reintroduced estrogen analogs after sex gland removal. Gorzek et al. [2], who observed a decrease from roughly 9.0 km·day^−1^ to less than 1.0 km·day^−1^ in a group of female C57BL/6J mice after ovariectomy and Hoskins, in 1925 [5] who observed a decrease from 15,142 rev·day^−1^ (≈ 14.6 km·day^−1^) in normal male rats to 3,283 rev·day^−1^ (≈ 3.2 km·day^−1^) in castrated male rats are just two examples of studies that have observed decreases in activity with removal of the sex steroids.

The preponderance of literature in this area has investigated the effect of estrogenic replacement on wheel running with some variability in post-surgical recovery of activity. Most recently, Gorzek et al. [2] observed recovery of wheel running activity to levels observed in control mice (≈ 85% compared to shams) of running distance in female mice with administered 17β-estradiol. Durrant [13] fed ovariectomized white rats a diet of glycerine prepared ovarian extracts and reported no effects on wheel revolution number suggesting limits to activity recovery with oral administration of steroids. Wang et al. [3] demonstrated a robust increase from less than 1,000 rev·day^−1^ to between 6,000 and 8,000 rev·day^−1^ in castrated male rats treated with ovarian tissue grafts from female littermates. This response equated to a 50–100% recovery compared to intact control animals. Bugbee and Simond [7] found 100% of wheel revolutions were recoverable with repeated injections of ovarian follicular fluid, but noted that when the dosage was tripled, additional improvements in activity were not observed. The designs and age of past experiments have made it difficult to determine dose responses and thus, it is unclear how the estrogen replacement dosages used in previous literature compare to that used in the current study. However, the various estrogen replacement protocols in the literature have resulted in a 50–100% recovery rate for activity with the lower recovery rates reported by these studies [2,3,7,13] similar to those observed in the present study.

### Potential androgenic effects

There are few studies regarding the androgenic influences on activity patterns in rodents available for comparison with the results of the current study. Before the discovery of testosterone, Hoskins [6] grafted testicular tissue into castrated rats, but did not observe changes in their activity patterns. In a similar study, Richter and Wislocki [33] used a more elaborate technique to introduce testicular grafts into male and female rats. The authors found a greater number of wheel revolutions in several of the animals and upon further histological investigations suggested that successful transplantation of the grafted tissue in most animals resulted in running at higher levels. Much later, Roy and Wade [9] investigated the effects of aromatizable testosterone propionate and found increased activity (from 2 km·day^−1^ to 4 km·day^−1^) in castrated male rats. To our knowledge, the data in the current study represent the first data available regarding the effect of testosterone replacement on activity patterns in both male and female mice and suggests that testosterone may play a larger role in regulating daily activity than previously suggested. Thus, our results suggest a broader picture of physical activity regulation by the sex steroids that also includes a yet to be outlined androgenic effect.

There are three lines of evidence commonly reported to support involvement of estrogen rather than testosterone compounds as the primary activity regulator in rodents. First, several experiments have shown a variable increase in activity in both male and female rodents when estrogenic compounds are delivered through a variety of administrative techniques [2–4,7,12,14,34–38]. Second, in studies of knockout mice of both sexes, Ogawa et al. [10] observed that activity levels were dependent upon an interaction between an estrogenic compound and estrogen receptor α. In their study, no differences in activity were seen after implantation of β-estradiol 3-benzoate in animals lacking the α isoform of the estrogen receptor. Third and most notable, Roy and Wade [9] observed increased activity patterns only with the administration of testosterone propionate, an aromatizable androgen and not with administration of dihydrotestosterone propionate, a non-aromatizable androgen in castrated rats [9].

**Figure 4:**
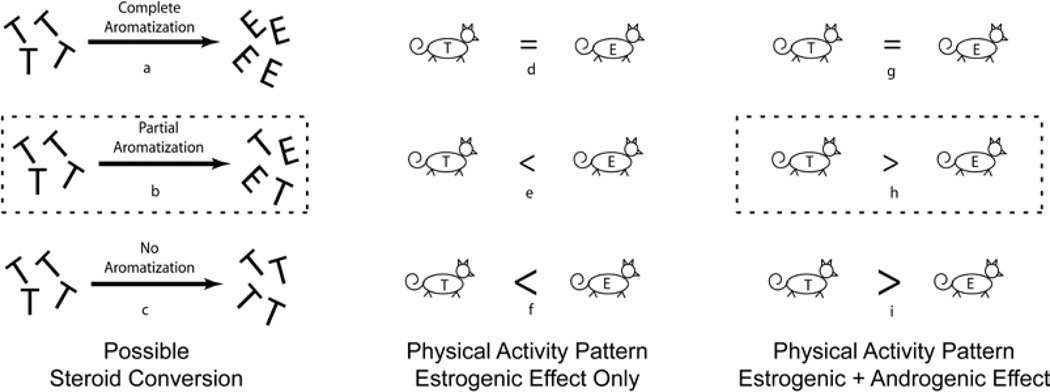
Schematic representation comparing mice receiving only 17β-estradiol (represented by mouse with ‘E’ on abdomen) to those receiving testosterone (represented by mouse with ‘T’ on abdomen) after surgical gonadectomy. Three scenarios (panels a–c) exist regarding testosterone’s conversion and lead to three possible wheel running responses if the estrogens are the primary effecters of activity (panels d–f) and three possible responses if an androgenic effect is also present (panels g–i). T=Testosterone, E=17β-estradiol, dotted outline=observed outcome from present dataset.

The mice in the present study that received 17β-estradiol capsules after gonadectomy would have elevated levels of the estrogens alone (Figure 4). Since the estrogens are not converted to testosterone, very little testosterone would be present in these mice. The mice receiving testosterone implants would fall into one of three categories regarding steroid levels in general circulation; 1) only estrogens are present (complete conversion of testosterone to estrogens via aromatization; Figure 4, panel a), 2) some of each steroid is present (some testosterone is aromatized to estrogens; Figure 4, panel b), and 3) only testosterone is present (no conversion to estrogens via aromatization; Figure 4, panel c).

Comparisons between the estrogen replacement group and the testosterone replacement groups mentioned above (no estrogen, some estrogen, and all estrogen) suggest the presence of an androgenic regulatory effect on physical activity (Figure 4). The precise dimensions of a silastic implant allowed a constant volume (approximately 200 to 300 µg·day^−1^ as suggested earlier) of steroid to be present in each capsule, which led to the release of similar amounts of steroids to all mice. Therefore, the circulating levels of estrogens in mice with 17β-estradiol capsules would be equal only to the levels of mice with testosterone capsules if the testosterone were converted completely to 17β-estradiol leaving no circulating testosterone (Figure 4, panels a, d, g). If the testosterone were completely converted to 17β-estradiol, it should be expected that the mice, regardless of steroid replaced, would not differ in activity performed. This was not the case in this study, as the mice receiving the testosterone capsules outperformed the mice receiving the 17β-estradiol capsules. In reality, replaced testosterone is probably not entirely converted to 17β-estradiol (Figure 4, panel b). The residual levels of testosterone remain available to interact in an androgenic manner that can affect activity levels. These observations conflict with the results of Roy and Wade [9] who suggested that the androgens needed to first be converted to estrogens prior to influencing wheel running; testosterone’s regulatory effects were not dependent upon estrogen.

### Other potential factors

The removal of the gonads, especially in female mice, not only removes the primary estrogen sources in these animals, but also a substantial progesterone source. While the influence of progesterone on wheel running activity was not the focus of this paper, past research has indicated a minimal effect of this steroid on activity regulation. Rodier [39] injected ovariectomized albino rats with 40 mg·kg^−1^·day^−1^ progesterone and found no changes to the activity pattern. In the same study, 8 mg·kg^−1^·day^−1^ of progesterone was given to intact female rats and was found to have a slight inhibitory effect [39]. Based on these lines of evidence, the effects of progesterone loss due to ovariectomy in the present study was likely minimal.

The changes observed in the body composition variables appear to relate to natural differences between the sexes and appeared to follow temporal patterns rather than patterns attributable to changes in steroid status. Past research [19] has shown changes in body composition characteristics with alterations in steroid levels; however, the brevity of the experimental periods in this study may have hindered full development of this effect. Furthermore, the effects of body mass on activity have been shown to be limited in non-obese rodents [22,23,40].

In conclusion, the current study investigated the influence of both testosterone and 17β-estradiol on physical activity patterns in male and female C57BL/6J mice. All three indices of activity were significantly decreased with removal of the sex steroids; running distance and duration were most responsive to alterations in circulating steroid levels. The differences in recovery of physical activity observed in mice implanted with 17β-estradiol (lower recovery) and testosterone (higher recovery) provided evidence that in mice, both steroids variably alter activity patterns. Based on the significantly larger magnitude of recovery with testosterone replacement, it is suggested that testosterone believed from limited past studies to only regulate activity via an estrogen dependent mechanism may also influence activity through a direct, androgenic mechanism.

## Figures and Tables

**Table 1:** Body mass and % body fat in male and female C57BL/6J mice with altered sex steroid concentrations during wheel running.

	Body Mass (g)			Body Fat (%)		
	Baseline	Gonadectomy	Replacement	Baseline	Gonadectomy	Replacement
*Males*						
Sham (n=3)	24.0 ± 2.7	23.8 ± 2.9	26.7 ± 4.5	11.65 ± 1.05	12.19 ± 0.53	11.98 ± 2.48
Testosterone (n=10)	24.1 ± 1.3	24.0 ± 0.7	25.6 ± 1.3	12.63 ± 1.11	12.53 ± 1.56	12.33 ± 0.78
17β-Estradiol (n=9)	24.9 ± 1.1	24.7 ± 1.1	28.2 ± 1.1	13.79 ± 3.47	12.92 ± 1.27	12.36 ± 0.73
*Females*						
Sham (n=3)	19.1 ± 0.6	18.5 ± 0.1	20.8 ± 0.5	13.43 ± 0.72	13.84 ± 0.94	12.51 ± 0.39
Testosterone (n=8)	19.7 ± 1.0	21.3 ± 1.2	23.6 ± 1.1	13.46 ± 1.15	12.79 ± 0.79	11.82 ± 0.76
17β-Estradiol (n=8)	18.9 ± 1.0	20.5 ± 1.2	23.4 ± 1.3	14.13 ± 1.86	13.11 ± 1.21	11.14 ± 0.51

Values are mean ± SD. Baseline data represents normal characteristics, gonadectomy data represents no circulating sex steroids, and replacement represents reintroduced sex steroids. No significant difference found for the sex by group by treatment interaction. Individual main effects were reported in the results section

**Table 2:** Sera/plasma testosterone and 17β-estradiol concentrations for male mice* at baseline and under various placebo and experiment conditions.

Condition (n=T/E_2_)	Testosterone (ng·ml^−1^) mean ± SD	17β-Estradiol (pg·ml^−1^) mean ± SD
Baseline (n=3/3)	6.3 ± 2.8	59.8 ± 16.7
Placebo (n=15/15)	11.5 ± 7.5	397.1 ± 881.5
	***Experimental***	
ORCH (n=3/2)	min	109.8 ± 29.7
ORCH+E_2_ (n=2/3)	0.2 ± 0.2	613.4 ± 185.9
ORCH+T (n=3/2)	15.4 ± 1.2	152.0 ± 53.2

* Sera/Plasma from 3 mice were pooled together to form a single data point (n); female blood samples were non-viable

**Abbreviations:** ORCH: Orchidectomy; E_2_: 17β-Estradiol Implant; T: Testosterone Implant; min: At the minimum of prediction curve
